# The quality and satisfaction of romantic relationships in transgender people: A systematic review of the literature

**DOI:** 10.1080/26895269.2020.1765446

**Published:** 2020-07-21

**Authors:** Ellen Marshall, Cris Glazebrook, Sally Robbins-Cherry, Serge Nicholson, Nat Thorne, Jon Arcelus

**Affiliations:** a Institute of Mental Health, Faculty of Medicine and Health Sciences, University of Nottingham, Nottingham, UK; b Nottingham Centre for Transgender Health, Nottinghamshire Healthcare NHS Foundation Trust, Nottingham, UK; c London, UK

**Keywords:** PRISMA, romantic relationships, support, systematic review, transgender

## Abstract

**Introduction:**

Romantic relationships are often a significant area of individuals’ lives and can have a positive impact on wellbeing. There is often a belief within society of romantic relationships ending upon the start of gender affirming transition, however this is often not reflected within clinical work or research studies. Despite this, currently not enough is known about romantic relationships for transgender individuals and their partners, and the impact gender affirming transition can have on the quality and satisfaction of these relationships.

**Aim:**

To critically and systematically review the available literature examining quality and satisfaction of romantic relationships for transgender individuals and their partners.

**Methods:**

Using PRISMA guidelines, major databases (Pubmed, PsycINFO and Web of Science) and relevant reference lists were searched for suitable articles up to January 2020. Each included article was assessed for methodological quality and the demographic data, methods and findings linked to relationship quality and satisfaction was extracted for analysis.

**Results:**

From 151 potentially relevant articles, 14 studies (six quantitative, eight qualitative) were included within the review. Most studies displayed moderate risk of bias due to cross-sectional designs and lack of reflexivity. Findings from quantitative studies suggest a bi-directional relationship between transition, relationship quality and satisfaction and wellbeing. Qualitative studies suggest transition can cause personal challenges for both transgender individuals and partners. Maintenance activities help buffer the impact of these challenges on relationship satisfaction and ensure positives are possible from relationships.

**Discussion:**

Gender affirming transition can impact on the quality and satisfaction of romantic relationships. Due to additional challenges transgender individuals and their partners may face, adequate support is required at personal, community and clinical level. There is a paucity of research in this area and current studies lack methodological rigor. Future research is essential to gain a further understanding of transgender relationships and the support required.

## Introduction

Studies have reported that only around half of romantic relationships for transgender individuals survive through gender affirming transition (Brown, 2009; Devor, 1997). This may be of concern to transgender individuals who are in the process of initiating gender affirming transition. However, in order to understand the real meaning of these findings a critical systematic review of the available literature is needed.

Romantic relationships are defined as relationships based on emotional and physical attraction, potentially leading to long-term intimate relationships (World Health Organization, 2017). They are often a hallmark and significant area of individuals’ lives. The quality and satisfaction of romantic relationships can be affected by many different factors such as age, individual’s self-esteem, personality, attachment type, among others (Erol & Orth, 2016; Heaton, 2002; Li & Chan, 2012; Malouff et al., 2010). Additionally, the benefits of being in a high quality and highly satisfied relationship on mental health and overall quality of life have been commonly reported for the general population (Simon, 2002). Research has shown that those who are in satisfied and high quality relationships report higher self-esteem (Luciano & Orth, 2017), improved wellbeing (Diener et al., 2008; Soons et al., 2009) and lower levels of anxiety and depression (Dush & Amato, 2005; Simon, 2002) than those who are not in a relationship.

Within the transgender population, research has consistently shown that treatment seeking transgender people who are not on cross sex hormone treatment report high levels of mental health problems, such as anxiety and depression (e.g., Arcelus et al., 2016; Bouman et al., 2017; Dhejne et al., 2016) and lower quality of life (Nobili et al., 2018a) compared to the general population. Within society transgender individuals often experience stigma and discrimination in many aspects of their lives which can limit both practical and social opportunities (White-Hughto et al., 2015). Using Meyer’s (2003) minority stress theory, the stigma and discrimination experienced by transgender individuals due to being a minority group within society may lead to negative self-appraisals and poor health outcomes, and may help to explain the high level of mental health problems reported (Hendricks & Testa, 2012).

Minority stressors may also impact on transgender individuals’ relationships and social life (Hendricks & Testa, 2012). Indeed, transgender individuals at all stages of transition (medical and social) report difficulties in interpersonal relationships (Nobili et al., 2018b; Stewart et al., 2018) and challenges within family dynamics (Dierckx et al., 2016). With regard to romantic relationships, transgender individuals may face challenges within several different aspects of relationships, including finding partners, disclosing to partners, and maintaining relationships (Platt & Bolland, 2017). Additionally, for partners, individuals may begin to question their own identity or sexual orientation (Meier et al., 2013) and can be over-whelmed by other transition related factors, including navigating new gender roles and relationship dynamics (Devor, 1997).

Currently there is a lack of research focusing onthe impact of both gender affirming transition and the minority stress associated with transition on romantic relationships for transgender individuals and their partners. With wider research showing the positive effect relationships can have on mental health within the general population (Loving & Slatcher, 2013), it is imperative to gain an understanding of the quality and satisfaction of transgender romantic relationships.

To the authors’ knowledge there has been no systematic review of the available literature exploring romantic relationships for transgender individuals and their partners. Previous reviewshave concentrated on certain aspects of the relationships, particularly sexual health and behavior (Becasen et al., 2019) with a predominant focus on HIV (Herbst et al., 2008; Operario et al., 2008). While these topics are essential for improving medical care, there is still a lack of overall understanding of romantic relationshipsfor transgender individuals and their partners.

In light of this, the aim of this review is to critically and systematically review the available literature examining the quality and satisfaction of romantic relationships for transgender individuals and their partners. It is hoped that by achieving this aim and focusing on the level of quality and satisfaction aspect of transgender relationships, the review will help to understand the impact of gender affirming transition, which is defined as both social and medical for this review, on romantic relationships and provide important information on how to support transgender individuals and their partners in the future.

## Methodology

### Eligibility criteria

The articles selected included all methodological designed research studies published in peer reviewed journals between January 1966 and January 2020 that explore romantic relationships (as defined in the introduction) for both transgender individuals and their partners. Studies describing other types of relationships (family, friends) were not included in this review. Additionally, studies were only eligible if they looked specifically at the quality and satisfaction of the romantic relationship.

In terms of study design, only research articles were considered, as opposed to discussion papers. See Table 1 for the full eligibility criteria.

**Table 1.** Eligibility criteria.

Category	Criteria
Study Population	*Included:* Transgender individuals Current or ex-partners of transgender individuals All races, ethnicities, and cultural groups *Excluded:* LGBT studies that do not describe transgender individuals as a separate category
Phenomena of Interest	*Included:* Articles examining romantic relationship quality and satisfaction *Excluded:* Articles focusing solely on sexual health or partner violence Articles examining relationships with any other family members
Setting / Context	All nations
Time period	Articles published from January 1966 to January 2020
Publication criteria	*Included:* Articles in print / peer reviewed literature *Excluded:* Articles in gray literature or non-peer-reviewed journals
Study design	*Included:* Qualitative and quantitative studies *Excluded:* Discussion papers Reviews or meta-analysis

### Search strategy

The systematic review adheres to the guidelines detailed in the PRISMA Statement (Moher et al., 2009). An electronic literature search was conducted between January 1966 and January 2020 using Pubmed, PsycINFO and Web of Science. Additionally, reference sections of identified articles and Google Scholar were examined for further relevant publications. The search used the following keywords: for terms referring to transgender individuals (transgender, transsexual, “gender transition”, “gender dysphoria”), and relationships (relationship, “romantic relationship”, “intimate relationship”, partner*, couple*, marriage). Every term used for transgender individuals was combined using the “OR” and the “AND” operate with every term used for relationships. Trans* was not used for this search as this obtained too many results, therefore the full term was used.

A second researcher (NT) completed an independent literature search using the same method described to further increase the validity of the search. Any papers that were identified by only one researcher were discussed between the two researchers and if there was no agreement, a third researcher was consulted. Once discussions were completed, both researchers (EM and NT) achieved the same numbers in the study search process.

### Study selection

**Figure 1.**
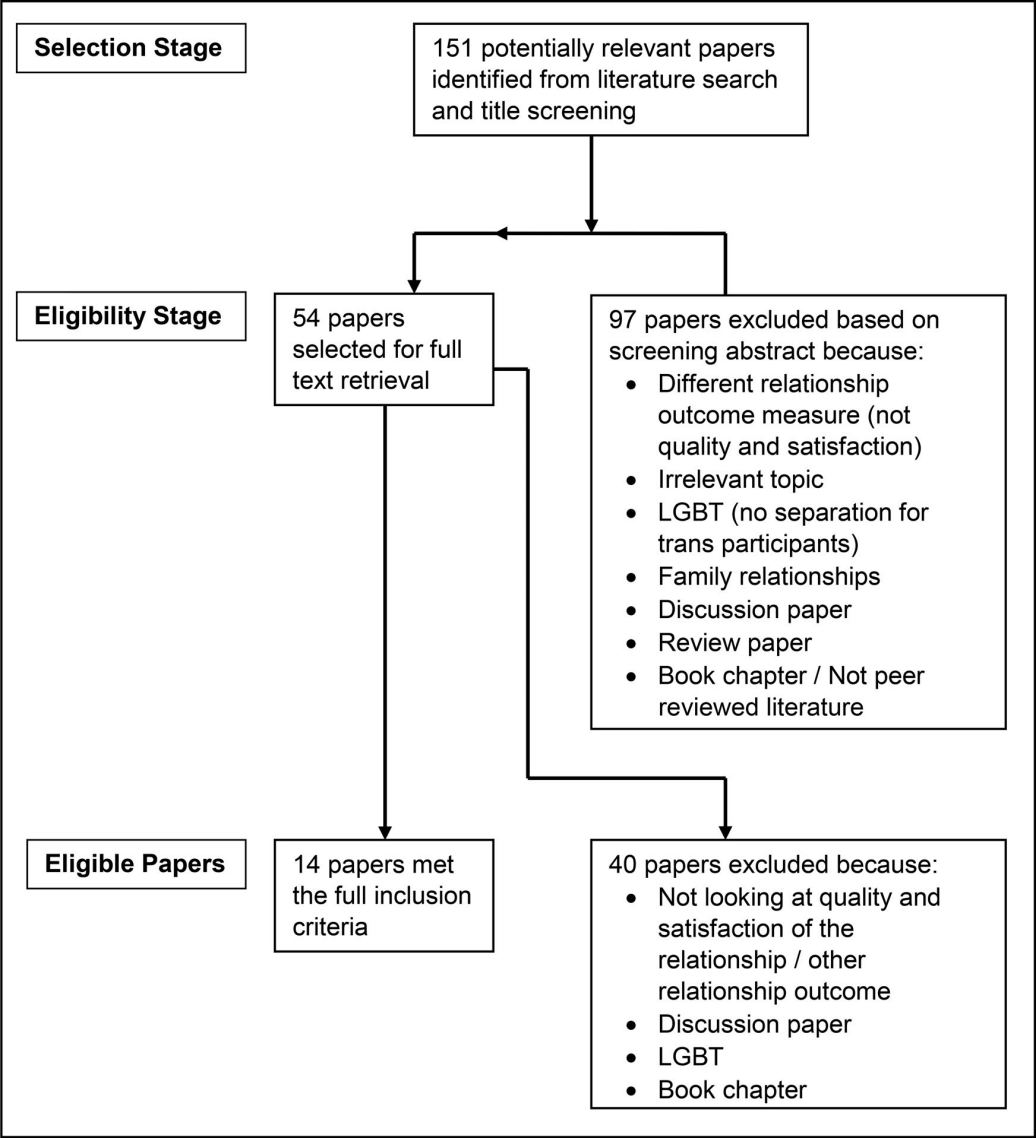
Study selection process.

As per PRISMA guidelines, studies were screened for eligibility in three stages: title, abstract and full text. In the first phase of screening, duplicates were removed, and the remaining titles were screened for eligibility by title for the present study (n = 151). This was then followed by screening the abstracts (n = 54), which was followed by full text screening which yielded 14 studies (see Figure 1). This final sample of 14 studies was discussed and agreed with the second researcher (NT) and were therefore included in the systematic review. Throughout the screening process the eligibility criteria was used to assess the appropriateness of the studies.

The main data extracted from the studies was different for the quantitative and qualitative studies. For the quantitative studies, the main data extracted was demographic data, tools used, and the main findings focused on the level of relationship quality and satisfaction. For qualitative studies, demographic information, key themes, and overall finings linked to quality and satisfaction of relationships was extracted.

### Quality assessment

To assess risk of bias in the qualitative studies an instrument adapted from Critical Appraisal Skills Program (CASP) Qualitative Research Checklist (2018) was used by the researchers. The instrument covered the most relevant criteria to assess risk of bias in qualitative studies. The items included in the instrument were divided up into sections based on the COREQ-32 checklist (Tong et al., 2007) which has been successfully used for reporting qualitative studies in medical research. This checklist allowed the researcher to assess the reliability and validity of the main findings, as well as assess the level of reflexivity and influence of researcher bias on the findings in order to gauge the overall methodological quality. To assess the risk of bias within the quantitative studies the CASP Cohort Study Checklist (2018) was used. This checklist focused on how representative the study was for the research aim and therefore allowed the researcher to determine the level of reliability for the research findings. Using each section on these checklists for the included studies allowed the researchers to award a final rating of good, fair or poor being awarded by the researchers.

## Findings

The following section will outline the main findings from the included studies. First the characteristics of all the studies will be discussed, followed by a description of the quantitative findings from the studies, and finally the description of the qualitative findings.

### Study characteristics

The oldest research article found in this area was published in 2006 (Hines, 2006) and the most recent articles was published in 2020 (Platt, 2020). As many studies looking at transgender health, most of the publications were from Western countries and predominantly from the USA (Alegria, 2010; Gamarel et al., 2014; Gamarel et al., 2019; Iantaffi & Bockting, 2011; Joslin-Roher & Wheeler, 2009; Meier et al., 2013; Pfeffer, 2008; Platt & Bolland, 2017, 2018). One study was from South Africa (Theron & Collier, 2013).

The majority of the studies (n = 8) were qualitative in nature, with most studies employing interviews as their main data collection method (Alegria, 2010; Iantaffi & Bockting, 2011; Joslin-Roher & Wheeler, 2009; Pfeffer, 2008; Platt & Bolland, 2017, 2018; Riggs et al., 2015; Theron & Collier, 2013). One study used in-depth semi-structured interviews and presented their findings in case studies (Hines, 2006). One qualitative study used online bulletin boards and chat room formats (Iantaffi & Bockting, 2011). Broadly, the qualitative studies explored the relationship experiences during the gender transition and the impact gender transition has on the relationship and the individuals involved.

The remaining six studies included in this review adopted a quantitative approach using a cross-sectional design and employing questionnaires to participants (Gamarel et al., 2014; Gamarel et al., 2019; Kins et al., 2008; Meier et al., 2013, Platt, 2020). The majority of them focused on relationship and individual factors influenced by the gender transition (Gamarel et al., 2014; Gamarel et al., 2019; Kins et al., 2008) and some assessed factors associated with relationship stability, quality and satisfaction during the transition (Meier et al., 2013; Platt, 2020).

Most of the studies were concerned with the quality and satisfaction of romantic relationships for transgender individuals (Hines, 2006;Iantaffi & Bockting, 2011; Kins et al., 2008; Meier et al., 2013; Platt & Bolland, 2017; Riggs et al., 2015). Some authors focused on the quality and satisfaction of the relationship for partners (Joslin-Roher & Wheeler, 2009; Kins et al., 2008; Pfeffer, 2008; Platt, 2020; Platt & Bolland 2018; Theron & Collier, 2013) and three studies researched couples together in the same study (Alegria, 2010; Gamarel et al., 2014; 2019). Details of all of the quantitative research articles included within this systematic review can be found in Table 2 and all qualitative studies can be found in Table 3.

**Table 2.** Quantitative studies exploring relationship experiences for transgender individuals and their partners.

Authors	Country (Year)	Aims	Population / Sample	Methods / Tools	Main Finding(s)	MQ¹
Kins et al.	Belgium (2008)	To compare the level relationship satisfaction between CW partnered with TM and CW partnered with CM.	N = 18 9 CW in a stable relationship with TM (who had undergone SRS, relationships had started at various stages of the transition) and 9 CW partnered with CM. Relationship duration ranged from 1 – over 10 years. Age range: 24 -40 Average age: 31.78	Self-constructed questionnaire Maudsley Marital Questionnaire (MMQ) Bem Sex Role Inventory (BSRI)	No significant difference in relationship satisfaction between CW partnered with TM and CW partnered with CM. No association between length of relationship, number of children, stage of relationship (i.e. dating, cohabitating, married) with relationship satisfaction for both CW partnered with TM and CW partnered with CM.	*
Meier et al.	USA (2013)	To assess relationship stability through transition and explore associations between mental health symptomology, social support and relationship stability.	N = 593 TM, various stages of transition. No requirement for relationship status or experience. Age range: 18 – 71 Average age: 27	Demographics Relationship status question (MSPSS) (DASS)	Half of relationships are maintained through transition. For the relationships that were formed pre transition and were no longer, 54% ended due to transition. The length of time since transition (measured by length of time on testosterone) was significantly longer for those whose relationships ended than for those who maintained a relationship. Participants in good quality and stable relationships report fewer symptoms of depression and anxiety and have better support to buffer against mental health symptomology.	**
Gamarel et al.	USA (2014)	To examine how transgender-related discrimination and relationship stigma are associated with relationship quality and mental health for TW and their CM partners.	N = 382 (191 couples) TW partnered with CM for at least 3 months. Average length of relationship = 37.9 months. Average age: 37.12 years Age range: Not reported	Demographics Dyadic Adjustment Scale (DAS) – adapted by authors Relationship stigma	Level of perceived discrimination for TW and CM was inversely correlated with relationship quality (higher discrimination – lower relationship quality) Age of CM partner was associated with relationship quality for TW and CM. Financial hardship was associated with relationship quality for TW and CM.	***
Riggs et al.	Australia (2015)	To explore relationship experiences and satisfaction in past relationship and assess the impact of satisfaction in past relationships on current or future relationships.	N = 160 119 trans participants, 41 gender diverse participants Average age: 39.8 years Age range: Not reported	Demographic questionnaire Open-ended questions – developed by research team	Gender differences reported - TW were more likely than TM or gender diverse people to experience challenges in their romantic relationships. Lower satisfaction in past relationships was negatively correlated with lower satisfaction in current and future relationships for both TW and TM.	*
Gamarel et al.	USA (2019)	To explore level of relationship commitment and associated factors of interpersonal stigma and psychological distress for TG individuals and their partners.	N = 382 (191 couples) TW partnered with CM for at least 3 months. Average length of relationship = 37.9 months. Average age: 37.12 years Age range: Not reported	Adapted Commitment subscale of the Triangular Theory of Love Scale	No significant difference in level of relationship commitment between TW and CM partner. For TW in relationships, relationship commitment is associated with interpersonal stigma, depressive and anxious symptoms. Level of relationship commitment buffers the relationship between interpersonal stigma and mental health symptoms for TW No significant associations were found for partners between interpersonal stigma, psychological distress and relationship commitment.	***
Platt	USA (2020)	To identify factors that predict relationship commitment among partners of transgender individuals.	N = 137 CW partners of self-identified transgender individuals (TM, TW & GD - all stages of transition). Average length of relationship = 5.14 years. Average age: 35 years Age range: Not reported	Demographic questionnaire Investment Model Scale (IMS) Brief Resilience Scale (BRS) Gender Role Beliefs Scale – Short version (GRBS-S) Attitudes Toward Divorce Scale	Personal resilience and years in the relationship prior to transition were associated with relationship commitment. Personal resilience was positively correlated with relationship commitment i.e. those who reported higher personal resilience also reported higher relationship commitment. Years in the relationship prior to transition was negatively correlated with relationship commitment i.e. those who had been together longer before transition reported lower levels of relationship commitment. Personal resilience and years in relationship before transition were associated with relationship satisfaction. Relationship satisfaction mediated the association between personal resilience and years in relationship before transition with relationship commitment.	**

¹ MQ: Methodological Quality (Instrument adapted from CASP Checklist, 2013).

* Poor;

** Fair;

*** Good.

TG: Transgender; TW: Trans women; TM: Trans men; GD: Gender diverse individual; CW: Cis women; CM: Cis men.

**Table 3.** Qualitative studies exploring relationship experiences for transgender individuals and their partners.

Authors	Country (Year)	Aims	Population / Sample	Methods	Themes	Main Finding(s)	MQ
Hines	UK (2006)	To explore how TG individuals negotiate their relationships through transition with partners and children.	N = 3 2 TW, 1 TM at various stages of transition. All are in relationships and live with their partners. Ages: 30, 40, 70	Narrative – semi-structured interviews / case studies	Emotional honesty	Participants focused on the importance of emotional honesty in the relationship process in order to negotiate challenges and maintain high satisfaction within the relationship during transition.	*
Pfeffer	USA (2008)	To explore the impact of gender transition on body image and the relationship for the cisgender partner.	N = 5 5 CW, lesbian partners of TM. Partners are at various stages of transition and relationship length ranges from one year to 11 years. Age range: 20-50	In-depth semi-structured interviews	No themes reported	Partner’s body image is impacted during their TG partner’s transition through internalization of external society and cultural norms and messages. This generally results in a negative body image which in turn has an impact on sexual intimacy and the overall quality of the romantic relationship.	*
Joslin-Roher and Wheeler	USA (2009)	To examine the experiences of lesbian, bisexual, and queer identified partners of TM through the transition process.	N = 9 LGB identified partners of trans men, 5 CW, 1 TW, 3 identify as female / femme. 7 participants currently in relationship with TM, 2 participants no longer with TM. Length of relationships ranged 6 months to 4 years. Age range: 22 – 36	Semi-structured interviews	Impact of transition on identity Community Caretaking Peer support The relationship itself Mental health	The LGB partner experience is complex and includes both challenges and rewards. Major theme reported was the impact of transition on the quality and satisfaction of the relationship. This included different aspects within the relationship including emotional and physical caretaking, sexuality, coming out, and sexual partnership.	**
Alegria	USA (2010)	To describe the relational dynamics that help sustain relationships for TW and their CW partners following disclosure.	N = 34 17 TW and their CW partners. TW were in various different stages of transition. Relationship duration ranged from 3 to 44 years. Age range: 30-69	Interviews and surveys	Challenges faced by couples: Sexual identity Relationship uncertainty Transition-related decision making Public presentation. Activities for maintaining relationship: Communication Self-talk Networks Positivity Impression management Social activism	TG individuals and their partners reported challenges within their relationships due to sexual identity and relationship uncertainty, transition-related decision making and public presentation. Participants reported activities that help to maintain relationships include communication, self-talk, networks, positivity, impression management and social activism. Challenges negatively impacted relationship satisfaction but maintenance factors help to buffer against this impact, and thus improve satisfaction.	**
Iantaffi and Bockting	USA (2011)	To explore the influence of heteronormativity, the related concept of sexual legitimacy, and gender as a binary construct on relationships for TG individuals.	N = 131 131 transgender participants *Part of larger quantitative study – only qualitative findings look at relationship quality. No additional information provided for the 131 sample for the interviews*	Online interview –bulletin boards and chat room formats	Four major themes emerged: Fearful of rejection Discomfort about disclosure and body Binary construct Society expectations	Findings suggest the quality and satisfaction of relationships for TG individuals’ relationships is influenced by heteronormative discourses within society. The discourse effect sexual intimacy which in turns impacts on the individual’s satisfaction within the relationship.	*
Theron and Collier	South Africa (2013)	To explore the relationship between cisgender female partners and trans persons.	N = 8 CW, all partnered with TM. 7 participants were currently in a relationship with TG individual, one participant’s relationship had ended. Partners were at varying stages of transition. Age range: 25-55	Semi-structured interviews	Three major themes identified: Sense of self for partners- sexual orientation and identity Co-transitioning process for partners Family and community acceptance of the trans-cis relationship	Relationship experiences of female partners of trans masculine individuals are diverse. Partners reported no change in their sexual orientation, experiencing their own transitioning experience and varied responses when disclosing to their own social circle. All of these impacted on their own overall experiences and their satisfaction with the relationship.	**
Platt and Bolland	USA (2017)	To examine the nature of partner relationships for people who identify as trans*.	N = 38 21 TW, 17 TM, various stages of transition. Not all participants were in a relationship. Age range: 18 - 70	Semi-structured interviews using ‘The Trans* Intimate Partner Experience Interview’	Gender binary system Disclosure Intimacy Communication, compromise, work on the relationship Live an authentic life	The relationship experience for TG individuals can be complex and filled with both joys and challenges. Social support, intimacy within the relationship and the binary system have a major impact on relationship quality and satisfaction. The disclosure process can be complex within long-term relationship. Communication and openness can be helpful with the challenges faced due to the disclosure process and overall satisfaction within the relationship.	**
Platt and Bolland	USA (2018)	To explore the unique elements of the experiences of those who partner with TG individuals.	N =21 13 CW, 2 CM, 4 TG, 2 gender fluid partnered with a TG individual. Partners were either TW (n = 8), TM (n = 9) or gender fluid (n = 4) and various stages of transition. Relationships had started at varying points of the transition. Age range: 20 – 61	Semi-structured interviews	Intimacy Sexual orientation labels Safety concerns Isolation Gender spectrum	Partners experience considerable change in their lives and relationships as their TG partner transitions. Challenges are faced, in particular through changes in intimacy, potential changes to personal identity labels, safety concerns and feelings of isolation as the partner. However, facing these challenges brings positives with partners experiencing a new appreciation of the gender spectrum and developing their own identity. All of which impacts on the partner perception of the quality and satisfaction of their relationship.	**

* MQ: Methodological Quality (Instrument adapted from CASP Checklist, 2013 and COREQ-32, 2007).

* Poor;

** Fair;

*** Good.

TG: Transgender; TW: Trans women; TM: Trans men; CW: Cis women; CM: Cis men.

### Quantitative findings

The six quantitative studies focused on both the level of relationship quality and satisfaction as well as the different factors associated with relationship quality and satisfaction for transgender individuals and their partners.

#### Level of relationship quality and satisfaction

The first five quantitative studies looked at the level of relationship quality and satisfaction for transgender individuals and their partners. The majority of the studies were descriptive and exploratory in nature and focused on different relationship outcome measures in order to gain an overall understanding of relationship quality and satisfaction. Focusing on the stability of relationships throughout the gender transition as a measure of relationship quality and satisfaction, Meier and colleagues (2013) reported the first and only prevalence rates for transgender relationships. From their USA based sample of 593 transgender men, they report half of relationships do survive through gender transition. For the relationships that were not maintained, 54% of the participants reported transition was the reason for the breakup. The authors also reportedthose in a relationship report lower levels of depression compared to single participants. Although the authors used a large sample within this study and recruited both through support groups and online, the majority of the sample (83%) were based in the USA and many (63%) were taking testosterone. The cultural differences between treatment pathways and society acceptance mean that it is difficult to generalize these results worldwide. Additionally, due to the inclusion of transgender men only, relationship stability through gender affirming transition for transgender women and any potential gender differences remains unknown.

The remaining five quantitative studies all looked at variables associated with relationship quality and satisfaction for the transgender individual and their partner. Transition (medical and social) can cause major changes within an individual’s own life and those around them. Therefore, it is not surprising that there is evidence that transition can have a major impact on romantic relationships for both individuals involved (Gamarel et al., 2014). In their study, Gamarel and colleagues reported couples experience specific minority stressors within their relationships and this negatively affected the relationship quality and mental health for both the transgender individual and the partner.These findings add an extra element to the understanding of relationship quality for transgender individuals as the participants reported both their own level and their perception of partner’s level of relationship quality. By using both measures the findings suggest the impact of minority stress on relationship quality occurs on both an individual and dyadic level, whereby partners who had experienced higher minority stress predicted lower relationship quality for their partner. Notably, their findings suggest that cisgender men’s perceptions of stigma about their transgender partner can influence the relationship and potentially lead to conflict for the couple. However, the cross-sectional design of this means that the authors are unable to determine causality between minority stress and relationship quality.

Also using a cross-sectional design but focusing on relationship quality and satisfaction for the partners, Platt (2020) explored predicting variables of relationship commitment and relationship satisfaction for cisgender individuals partnered with a self-identified transgender individual. They reported the only two significant variables within their study were personal resilience and the number of years in the relationship prior to transition. Both these variables were associated with relationship satisfaction and relationship satisfaction also mediated the relationship between these variables and relationship commitment, thus emphasizing the importance of relationship satisfaction in the overall relationship experience for partners during transition. While this study was exploratory in nature, the authors were obviously unable to include all variables that may be associated with relationship commitment. Therefore, while the authors included an impressive number of variables within their analysis, it must be noted other variables may be involved with relationship satisfaction and relationship commitment and further exploratory research is required in order to assess other predictive variables.

Interestingly when comparing transgender relationships (defined as a transgender man and a cisgender women by the authors), with a cisgender, heterosexual couple, Kins and colleagues (2008) reported no difference of relationship satisfaction. These findings suggest transition does not have an impact on relationship satisfaction, however there is concern regarding the sample used in this study. None of the participants recorded scores considered ‘low’ on the MMQ, thus implying all of the couples that took part were relatively satisfied with their relationship which does not reflect the general population (Hendriks et al., 1991). Furthermore, the small sample size of nine results in a lack of confidence for generalizing these findings and provides a potential explanation for why no associations were found between the associated variables and relationship satisfaction in the second part of study. However, the study does provide a foundation for future studies to use.

#### Impact of relationships on transition

When focusing on factors that may affect the relationship quality and satisfaction, it is also evident there is potentially a bi-directional relationship between transition and the level of relationship quality and satisfaction for transgender individuals and their partners. In particular, studies have also looked at the impact of relationship quality on transition for both the transgender individual and their partner on an individual level. Using relationship commitment as their outcome measure for transgender relationships, Gamarel and colleagues (2019) expanded on the findings from their 2014 study and examined the associations between interpersonal stigma, psychological distress and relationship commitment for transgender couples (defined as one transgender individual and one cisgender individual by the authors). Using an adapted form of the Commitment subscale for the Triangular Theory of Love Scale, the participants showed no difference in the reported level of relationship commitment between transgender individuals and partners. As the authors used an adapted scale it is not possible to gauge the level of commitment using the raw scores reported or compare to other studies in order to assess the relative level of relationship commitment for the study sample. Additionally, couples showed relationship commitment can reduce the associations between interpersonal stigma and psychological distress. This finding was only significantly shown for the transgender individual only, not their partner, thus suggesting the impact of relationship commitment on an individual’s wellbeing differs between transgender women and their cisgender male partners.

Despite the methodological limitations and gaps that are still in the literature, the overall message of the above studies suggest transition can negatively impact on relationship stability and overall relationship quality and satisfaction.

### Qualitative findings

Using a qualitative methodology, researchers have explored transgender romantic relationships further and have expanded upon on the quantitative findings within this area. Researchers have tended to focus upon the quality and satisfaction of the relationship either for the transgender individual or for the partner and look at the experience as a whole for each individual. In the only qualitative study that researched couples together, Alegria (2010) explored the relational dynamics that help to sustain relationships for transgender women and their cisgender female partners and thus improve relationship quality and satisfaction. The authors reported it can be very challenging for couples to navigate feelings of uncertainty about their relationship and their sexual identities in addition to experiencing difficulties with making decisions about transition and how they will present in public spaces. In order to deal with these challenges, and thus reduce the negative impact onto their level of satisfaction with the relationship, the couples described the importance of communication, self-talk and forming strong personal networks. The couples explained these maintenance activities helped buffer the negative impact of the challenges, and thus help to improve their overall satisfaction with the relationship. By researching the couple together and seeing both sides of the relationship provides an in-depth insight into the dynamics of the relationship and thus provides essential information to the understanding of transgender relationships. Furthermore, the relatively large sample size (n = 17 couples) for qualitative research ensured a large amount of new information and understanding was gained. However, all of the couples in the study had been together a relatively long-time (over 3 years) and all but one couple were married. Study findings may be different for couples who have been together for shorter time or are not married and therefore the findings cannot be generalized to all relationships.

#### Transgender experiences

When looking at relationships solely for transgender individuals, qualitative studies found a range of factors associated with the quality and satisfaction of relationships. Taking a sociological approach Hines (2006) found emotional honesty and to be an important part of the relationship experience for transgender individuals. The participants reported that often the intimate aspects of the relationship ended but their caring and closeness with each other continued which helped maintain the quality and satisfaction within their relationships. The case study methodology does result in a lack of diversity within the study and therefore the findings cannot be drawn wider. However, the themes reported do provide a unique and insightful insight into maintaining quality and satisfaction within transgender relationships.

Focusing on potential challenges affecting relationship quality for transgender individuals, Iantaffi and Bockting (2011) carried out a large mixed methods study in the USA. Within the interview aspect of the study the participants discussedthe difficulties within their relationship were predominantly due to heteronormative discourses and binary constructs within society. The participants’ perceived rigid gender role beliefs subsequently had a negatively impact on their own self-esteem, which in turn impacted on their level of satisfaction with the relationship. However, the findings must be used with caution as while there was a large sample within Iantaffi and Bockting’s study for the survey element, there is a significant reduced number interviewed and limited information available for these participants. Therefore, it is difficult to apply these findings to all transgender relationships. Furthermore, the participants were recruited for interview through self-referral which may evoke selection bias.

Taking a broader approach and looking at the overall experience of relationships for transgender individuals, Platt and Bolland (2017) reported similar findings to previous studies with participants discussing the challenges of disclosure and the gender binary system within society. However, as the authors took an open approach to the research and looked at the relationship as a whole, they found participants also discussed the feeling of wanting to live an authentic life and the importance of communication, compromise and work required within the relationship. These relatively novel themes provide information for the positives possible from the relationship as well as how transgender individuals may be best supported with their relationships, thus adding to the understanding of relationship quality and satisfaction for transgender individuals. The sample within this study was diverse with 21 transgender women and 17 transgender men, however any differences within the sample were not discussed within the study. Furthermore, information regarding stage of transition and any treatment received for the participants was not provided. As there is strong evidence of improved social functioning following social transition (Johansson et al., 2010; Murad et al., 2010) it can be hypothesized romantic relationships will change through transition. Therefore, while it is assumed the findings from Platt and Bolland’s study can relate to all stages of transition due to the diversity in their sample, this will need to be investigated further.

#### Partner experiences

In addition to looking at the quality and satisfaction of relationships for transgender individuals, recent studies have focused on partners and their level of relationship quality satisfaction within their relationship. These studies tend to take a broad approach and look at the partner experience and the impact this experience has on the level of quality and satisfaction.

Firstly, focusing on one particular aspect of the experience for partners, several studies looked at the impact of transition at an individual level. Pfeffer (2008) found that within their sample of five cisgender female partners of transgender men, the participants reported their partner’s transition often led to their own internal questioning around sexuality and identity and affected their body image. While it is previously been found that transgender individual’s body image generally improves through the transition process (Owen-Smith et al., 2018) it is surprising the partner body image can also be affected. This may be due to the transition increasing the spotlight on particular body parts. As their partner is particularly dysphoric about certain parts of their body, this may cause increased awareness for the partners about their own body. A negative body image has strong implications for psychosocial adjustment and social functioning (Green & Pritchard, 2003), and therefore the findings can be used to show an association between being partnered with a transgender individual and their own, individual wellbeing. However, the study is limited by its relatively small and non-diverse sample and the direct focus of the study may have resulted in essential information relating to the quality and satisfaction of the relationship for the partner being lost.

Internal questioning and exploration of identity was also found to be an important part of the relationship experience for the participants in Joslin-Roher and Wheeler (2009) study. One main theme reported by the authors was the impact of transition on the relationship itself. Within this theme the participants discussed many different aspects of the relationship that were affected by their partners’ transition and these all impacted on their own wellbeing and their level of satisfaction within the relationship. These aspects included understanding their own sexuality and using external labels for their sexuality, changes within sexual intimacy and support and reactions form others. Gender roles were also a major influencer on relationship quality and satisfaction for partners and the participants described often taking on a caretaking role which they often found challenging.

Looking at the whole experience of being partnered with a transgender individual and the impact this experience has on relationship quality and satisfaction, Theron and Collier (2013) explored relationships for cisgender partners of transmen in South Africa. Similar to earlier partner studies, participants described experiencing their own transition by developing personally as their partner transitions. The authors termed this the ‘co-transitioning experience’. In an impressive follow-up study, Platt and Bolland (2018) were able to expand of Theron and Collier’s findings and add more understanding to the ‘co-transitioning experience’. The authors reported similar themes and then added that the participants reported these aspects of the relationship and the transition itself often lead to feelings of loneliness and isolation which negatively influencing their relationship satisfaction. Additionally, the participants noted the importance of communication to ensure a healthy relationship which in turn improved their relationship quality and satisfaction. Furthermore, similarly to many of the previous qualitative studies reported, the participants noted the changes within sexual intimacy due to the transition and this also impacted in their perception of the relationship quality. This study is the most diverse in terms of its sample out of all the partner studies with all cisgender females, cisgender males, transgender individuals and gender diverse individuals partnered with transgender men, transgender women and gender-diverse individuals taking part. The differences and range of experiences reported by the participants reflects the diversity in the sample.

In summary, similarly to the quantitative findings, the qualitative studies included in this review generally report transition does impact on relationship quality satisfaction. The study reports generally transition poses challenges to both the transgender individual and the partner which negatively impacts on the level of relationship quality and satisfaction. However, expanding on this, the findings show there are maintenance techniques such as communication and support networks which individuals can use to buffer against this impact, and help maintain and potentially improve relationship quality and satisfaction.

## Discussion

The aim of this review was to critically and systematically review the available literature examining transgender romantic relationships in order to understand the effect gender affirming transition has on the quality and satisfaction of relationships for transgender individuals and their partners. Overall, the review found there is limited information on romantic relationships for transgender individuals and their partners. In particular there is a paucity of quantitative studies and all studies within this area lack a certain level of methodological quality, therefore any findings reported are difficult to generalize and use within clinical settings. Furthermore, as society continues to move forward and gender identity is becoming more fluid, it is imperative to gain an understanding of all transgender relationships through larger studies in order to understand how to support individuals in the future.

The review found that the rate of relationships for transgender individuals through transition is currently unknown. It is reported 50% of relationships end for transgender men (Meier et al., 2013) but there are no rates reported for transgender women. However, studies have found transgender men are more likely to be in a relationship than transgender women (Riggs et al., 2015). It is hypothesized that this difference may be due to the higher level of stigmatization within society reported by transgender women (Baams et al., 2013; Roberts et al., 2013), which may prevent transgender women from beginning romantic relationships due to the fear of disclosure and reactions from potential partners. Despite these gender differences, both transgender men and transgender women have been found to have significantly less sexual and romantic experiences compared to cisgender peers, despite appearing to fall in love at similar rates (Bungener et al., 2017). These findings indicate an association between transition and being in a relationship and thus suggests gender affirming transition can have an impact on the level of relationship quality and satisfaction, perhaps as an additional consequence of the minority stress experienced by transgender individuals (Meyer, 2003).

The review also found both transgender individuals and their partners face a number of challenges within their relationships, all of which affect relationship quality and satisfaction (e.g., Alegria, 2010; Platt & Bolland, 2017, 2018). These difficulties include the challenges of disclosure, society’s binary and heteronormative norms and expectations, and internal questioning and negotiation provoked by the transition (Iantaffi & Bockting, 2011; Joslin-Roher & Wheeler, 2009). Interestingly, it appears these challenges impact on an individual level first by affecting personal wellbeing and causing internal questioning, which then impacts on the perceived level of quality and satisfaction of the relationship. These findings add to the understanding of transgender relationships and emphasize the need for personal support for this population. Focusing on the impact of societal norms and expectations on the relationship, this particular finding adds to the minority stress theory (Meyer, 2003), whereby it appears transgender individuals and their partners face extra pressure within their relationship due to societal norms. As it is evidenced minority stress can lead to increased psychological distress (Hendricks & Testa, 2012) it is important for this element of minority stress to be understood and explored further in order to help decrease the impact of the stressors in the future.

Overall this review found there is an apparent need for support for transgender individuals and their partners for their romantic relationships. This is due to the challenges reported within the research studies and the positives impact relationships can have on wellbeing that was also reported (Meier et al., 2013). This support can be provided by clinicians, community support groups and those with personal relationships to the individuals. On a clinical level, clinicians will need to be aware of the possibility of transgender individuals having less relationship and sexual experiences than cisgender individuals. In particular, due to the high amount of younger people attending gender clinics (Holt et al., 2016) it would be beneficial for the clinicians to be aware many transgender individuals will not have any relationship or sexual experience prior to starting their social or medical transition. Therefore, the increased stress and anxiety that may be caused by individuals negotiating their first relationship or sexual experience must be taken into consideration when working with service users. Furthermore, clinicians working with younger service users must be aware of the diversity younger transgender individuals may experience within their relationships. As society becomes more fluid, it is important to recognize the value of any relationship experience an individual may have, for example, online or in virtual form, even if not perceived as currently conventional or heteronormative. Any experience may affect their wellbeing and future relationships, and this should not be overlooked.

Clinicians may also be able to help with maintaining relationships through transition. This review found emotional honesty and communication are important maintenance techniques within relationships for transgender individuals and their partners. These findings echo those from cisgender studies, with those who report higher levels of honesty in the relationship show higher levels of trust, leading to feeling highly satisfied with their relationship (Bello et al., 2008). Therefore, clinicians should aid couples in increasing their communication and honesty within the relationship. Focusing on the partner experience, clinicians must remember transition is not an individual process and all those involved can go through their own transition experience. It has been found by sharing similar experiences with others and meeting others in similar situations can help in other domains such as mental health, carers (Fung & Chien, 2002; Pistrang et al., 2008). With this in mind, clinicians should appropriately signpost partners to community support groups to engage with other partners with aim of trying to reduce the feelings of isolation and loneliness currently reported by partners. Communication with the community and service users would also be beneficial to ensure clinicians are aware of the best and most effective support available and thus are able to signpost in the most appropriate directions. Based on the research findings showing relationship quality and satisfaction can be impacted at all stages of gender affirming transition, it is imperative for clinicians to offer all of the aforementioned support at all stages of transition.

On a more general level of support, the findings from Meier and colleagues (2013) suggested being in a relationship during transition can help improve overall wellbeing for transgender individuals and thus show the importance of partner support for improving transgender individuals’ wellbeing. These findings are particularly of note for individuals who do not access medical treatment as they show how a romantic partner can help during social transition or when the transgender individual is not able to access treatment (i.e. due to long waiting lists). Furthermore, as it appears couples do not use dyadic coping through transition it is imperative for the partner to have their own social support through transition while they are supporting their transgender partner. Community groups and online forums and websites would be able to facilitate this.

Despite the important findings gained from the research studies, the review was limited by the methodological quality of studies considered within it. Firstly, due to cross-sectional designs in quantitative studies and a lack of information regarding the participants and convenience sampling in qualitative studies it is difficult to describe how relationships progress and how relationship quality and satisfaction change (if at all) through the transition. Disclosure and the first conversations regarding gender identity are a major part of transition and can have a major impact on relationship quality and satisfaction (Bethea & McCollum, 2013). Going through these major events as a couple may be different to that of couples who begin a relationship after the transgender individual has undergone gender affirmative medical treatment. Based on the studies included in this review it is currently not possible to determine if what has been reported in the included studies can be expanded to include all transgender individuals and relationship quality and satisfaction at all stages of transition. Studies adopting a longitudinal study design are required in order to understand relationships throughout transition and thus be able to provide the most effective support.

Furthermore, presumably due to the difficulties of recruiting within this area, often studies overlook certain demographics. In particular, there is an evident lack of cisgender male or male identifying participants in the partner studies. Due to the possibility of gender differences for these experiences (Riggs et al., 2015), it would be helpful for future research to include all demographics. This should also include all ages and ethnicities too. It also must be noted despite the main findings from many of the qualitative studies in this review reporting the difficulties of binary constructs, often the studies undertook the research with a binary outlook assessing relationships between a transgender relationship and a cisgender partner (e.g., Alegria, 2010). While these studies provide interesting and useful findings, future research would benefit from taking a more gender diverse approach and assessing all relationship and dyad possibilities.

There is no validated measure used within the quantitative studies and therefore it is not possible to determine the level of relationship quality and satisfaction for transgender romantic relationships. It is also not possible to compare to alternative data sets as the measures used are often amended by the authors to fit their study population. In order to improve the understanding of transgender romantic relationships it is imperative a measure is created and used exclusively within this research area. Additionally, the research area of romantic relationships generally is broad and often a multi-faceted concept. While interesting this does present some difficulties in research as often different aspects of relationships are focused on within studies. For example, although this review is examining the quality and satisfaction of relationships, many of the studies looked at other measures of relationships, such as commitment and stability and linked these back to quality and satisfaction. While the findings of these studies are important in understanding transgender relationships, it would be useful for all further research to clearly define their research focus and the outcome of the relationship they will be assessing. This will help to bring all the research in this area together and add to overall understanding of transgender relationships.

In addition to the methodological limitations for the included studies, the current systematic review also has some limitations which should be considered. This review excluded studies that explored partner violence and sexual health. While these can be major aspects of relationships, due to the large number of studies within these areas they warrant their own reviews. Therefore, the findings from this review cannot be used to describe or explain these areas of relationships.

In summary, this review found gender affirming transition can impact on the quality and satisfaction of romantic relationships for transgender individuals and their partners. Both transgender individuals and partners face a number of challenges within the relationship, but positives are possible from the relationship and dissolution is not inevitable as may have been previously perceived. Due to the additional challenges transgender individuals and their partners may face, adequate support is required at both personal, community and clinical level. There is currently a paucity of research in this area and the current studies lack methodological rigor. Future research is essential to gain a further understanding of transgender relationships and the support required. It is hoped this review can be used as a foundation for this future research.

## References

[CIT0001] Alegria, C. A. (2010). Relationship challenges and relationship maintenance activities following disclosure of transsexualism. *Journal of Psychiatric and Mental Health Nursing*, *17*(10), 909–916. doi: 10.1111/j.1365-2850.2010.01624.x 21078006

[CIT0002] Arcelus, J., Claes, L., Witcomb, G. L., Marshall, E., & Bouman, W. P. (2016). Risk factors for non-suicidal self-injury among trans youth. *The Journal of Sexual Medicine*, *13*(3), 402–412. doi: 10.1016/j.jsxm.2016.01.003 26944465

[CIT0003] Baams, L., Beek, T., Hille, H., Zevenbergen, F. C., & Bos, H. M. (2013). Gender nonconformity, perceived stigmatization, and psychological well-being in Dutch sexual minority youth and young adults: A mediation analysis. *Archives of Sexual Behavior*, *42*(5), 765–773. doi: 10.1007/s10508-012-0055-z 23358856

[CIT0004] Becasen, J. S., Denard, C. L., Mullins, M. M., Higa, D. H., & Sipe, T. A. (2019). Estimating the prevalence of HIV and sexual behaviors among the US transgender population: A systematic review and meta-analysis, 2006–2017. *American Journal of Public Health*, *109*(1), e1–e8. doi: 10.2105/AJPH.2018.304727 PMC6301428 30496000

[CIT0005] Bello, R. S., Brandau-Brown, F. E., & Ragsdale, J. D. (2008). Attachment style, marital satisfaction, commitment, and communal strength effects on relational repair message interpretation among remarrieds. *Communication Quarterly*, *56*(1), 1–16. doi: 10.1080/01463370701838968

[CIT0006] Bethea, M. S., & McCollum, E. E. (2013). The disclosure experiences of male-to-female transgender individuals: A systems theory perspective. *Journal of Couple & Relationship Therapy*, *12*(2), 89–112. doi: 10.1080/15332691.2013.779094

[CIT0007] Bouman, W. P., & Arcelus, J. (2017). *The transgender handbook: A guide for transgender people, their families, and professionals*. Nova Publishers.

[CIT0008] Bouman, W. P., Claes, L., Brewin, N., Crawford, J. R., Millet, N., Fernandez-Aranda, F., & Arcelus, J. (2017). Transgender and anxiety: A comparative study between transgender people and the general population. *International Journal of Transgenderism*, *18*(1), 16–26. doi: 10.1080/15532739.2016.1258352

[CIT0009] Brown, N. R. (2009). “I’m in transition too”: Sexual identity renegotiation in sexual-minority women’s relationships with transsexual men. *International Journal of Sexual Health*, *21*(1), 61–77. doi: 10.1080/19317610902720766

[CIT0010] Bungener, S. L., Steensma, T. D., Cohen-Kettenis, P. T., & de Vries, A. L. (2017). Sexual and romantic experiences of transgender youth before gender-affirmative treatment. *Pediatrics*, *139*(3), e20162283. doi: 10.1542/peds.2016-2283 28242863

[CIT0011] Critical Appraisal Skills Programme. (2018). *CASP Qualitative Research Checklist*. https://casp-uk.net/wp-content/uploads/2018/01/CASP-Qualitative-Checklist-2018.pdf

[CIT0059] Critical Appraisal Skills Programme. (2018). *CASP Cohort Study Checklist*. https://casp-uk.net/wp-content/uploads/2018/01/CASP-Cohort-Study-Checklist_2018.pdf

[CIT0012] Devor, H. (1997). *FTM: Female-to-male transsexuals in society*. Indiana University Press.

[CIT0013] Dhejne, C., Van Vlerken, R., Heylens, G., & Arcelus, J. (2016). Mental health and gender dysphoria: A review of the literature. *International Review of Psychiatry ( Psychiatry)*, *28*(1), 44–57. doi: 10.3109/09540261.2015.1115753 26835611

[CIT0014] Diener, M. L., Diener McGavran, M. B., Eid, M., & Larsen, R. J. (2008). What makes people happy? A developmental approach to the literature on family relationships and well-being. In M. Eid & R.J. Larsen (Eds.), *The science of subjective well-being* (pp. 347–375). Guilford Press.

[CIT0015] Dierckx, M., Motmans, J., Mortelmans, D., & T'sjoen, G. (2016). Families in transition: A literature review. *International Review of Psychiatry ( Psychiatry)*, *28*(1), 36–43. doi: 10.3109/09540261.2015.1102716 26618401

[CIT0016] Dush, C. M. K., & Amato, P. R. (2005). Consequences of relationship status and quality for subjective well-being. *Journal of Social and Personal Relationships*, *22*(5), 607–627. doi: 10.1177/0265407505056438

[CIT0017] Erol, R. Y., & Orth, U. (2016). Self-esteem and the quality of romantic relationships. *European Psychologist*, *21*(4), 274–283. doi: 10.1027/1016-9040/a000259

[CIT0019] Fung, W. Y., & Chien, W. T. (2002). The effectiveness of a mutual support group for family caregivers of a relative with dementia. *Archives of Psychiatric Nursing*, *16*(3), 134–144. doi: 10.1053/apnu.2002.32951 12037799

[CIT0020] Gamarel, K. E., Reisner, S. L., Laurenceau, J. P., Nemoto, T., & Operario, D. (2014). Gender minority stress, mental health, and relationship quality: A dyadic investigation of transgender women and their cisgender male partners. *Journal of Family Psychology : Psychology)*, *28*(4), 437–447. doi: 10.1037/a0037171 PMC4122619 24932942

[CIT0021] Gamarel, K. E., Sevelius, J. M., Reisner, S. L., Coats, C. S., Nemoto, T., & Operario, D. (2019). Commitment, interpersonal stigma, and mental health in romantic relationships between transgender women and cisgender male partners. *Journal of Social and Personal Relationships*, *36*(7), 2180–2201. doi: 10.1177/0265407518785768 31086428 PMC6510026

[CIT0022] Green, S. P., & Pritchard, M. E. (2003). Predictors of body image dissatisfaction in adult men and women. *Social Behavior and Personality: An International Journal*, *31*(3), 215–222. doi: 10.2224/sbp.2003.31.3.215

[CIT0023] Heaton, T. B. (2002). Factors contributing to increasing marital stability in the United States. *Journal of Family Issues*, *23*(3), 392–409. doi: 10.1177/0192513X02023003004

[CIT0024] Hendriks, A. A. J., Sanderman, R., & Ormel, J. (1991). Value of the Maudsley Marital Questionnaire (MMQ) as a measure for quality of the partner relationship: A multitrait-multimethod and confirmatory factor analysis (De waarde van de Maudsley Marital Questionnaire (MMQ) alsmaatvoor de kwaliteit van de partnerrelatie: eenmultitrek-multimethodenconfirmerendefactoranalyse). *NederlandsTijdschrift Van de Psychologie*, *46*, 187–195.

[CIT0025] Hendricks, M. L., & Testa, R. J. (2012). A conceptual framework for clinical work with transgender and gender nonconforming clients: An adaptation of the Minority Stress Model. *Professional Psychology: Research and Practice*, *43*(5), 460–467. doi: 10.1037/a0029597

[CIT0026] Herbst, J. H., Jacobs, E. D., Finlayson, T. J., McKleroy, V. S., Neumann, M. S., & Crepaz, N, & HIV/AIDS Prevention Research Synthesis Team. (2008). Estimating HIV prevalence and risk behaviors of transgender persons in the United States: A systematic review. *AIDS and Behavior*, *12*(1), 1–17. doi: 10.1007/s10461-007-9299-3 17694429

[CIT0027] Hines, S. (2006). Intimate transitions: Transgender practices of partnering and parenting. *Sociology*, *40*(2), 353–371. doi: 10.1177/0038038506062037

[CIT0028] Holt, V., Skagerberg, E., & Dunsford, M. (2016). Young people with features of gender dysphoria: Demographics and associated difficulties. *Clinical Child Psychology and Psychiatry*, *21*(1), 108–118. doi: 10.1177/1359104514558431 25431051

[CIT0029] Iantaffi, A., & Bockting, W. O. (2011). Views from both sides of the bridge? Gender, sexual legitimacy and transgender people’s experiences of relationships. *Culture, Health & Sexuality*, *13*(3), 355–370. doi: 10.1080/13691058.2010.537770 PMC3076785 21229422

[CIT0030] Johansson, A., Sundbom, E., Höjerback, T., & Bodlund, O. (2010). A five-year follow-up study of Swedish adults with gender identity disorder. *Archives of Sexual Behavior*, *39*(6), 1429–1437. doi: 10.1007/s10508-009-9551-1 19816764

[CIT0031] Joslin-Roher, E., & Wheeler, D. P. (2009). Partners in transition: The transition experience of lesbian, bisexual, and queer identified partners of transgender men. *Journal of Gay & Lesbian Social Services*, *21*(1), 30–48. doi: 10.1080/10538720802494743

[CIT0032] Kins, E., Hoebeke, P., Heylens, G., Rubens, R., & De Cuypere, G. (2008). The female-to-male transsexual and his female partner versus the traditional couple: A comparison. *Journal of Sex andMarital Therapy*, *34*(5), 429–438. doi: 10.1080/00926230802156236 18770112

[CIT0033] Li, T., & Chan, D. K. S. (2012). How anxious and avoidant attachment affect romantic relationship quality differently: A meta‐analytic review. *European Journal of Social Psychology*, *42*(4), 406–419. doi: 10.1002/ejsp.1842

[CIT0034] Loving, T. J., & Slatcher, R. B. (2013). Romantic relationships and health. In J. A. Simpson & L. Campbell (Eds), *The Oxford handbook of close relationships* (pp. 617–637). Oxford University Press.

[CIT0035] Luciano, E. C., & Orth, U. (2017). Transitions in romantic relationships and development of self-esteem. *Journal of Personality and Social Psychology*, *112*(2), 307–328. doi: 10.1037/pspp0000109 27379474

[CIT0036] Malouff, J. M., Thorsteinsson, E. B., Schutte, N. S., Bhullar, N., & Rooke, S. E. (2010). The five-factor model of personality and relationship satisfaction of intimate partners: A meta-analysis. *Journal of Research in Personality*, *44*(1), 124–127. doi: 10.1016/j.jrp.2009.09.004

[CIT0037] Meier, S. C., Sharp, C., Michonski, J., Babcock, J. C., & Fitzgerald, K. (2013). Romantic relationships of female-to-male trans men: A descriptive study. *International Journal of Transgenderism*, *14*(2), 75–85. doi: 10.1080/15532739.2013.791651

[CIT0038] Meyer, I. H. (2003). Prejudice, social stress, and mental health in lesbian, gay, and bisexual populations: conceptual issues and research evidence. *Psychological Bulletin*, *129*(5), 674–697. doi: 10.1037/0033-2909.129.5.674 12956539 PMC2072932

[CIT0039] Moher, D., Liberati, A., Tetzlaff, J., & Altman, D. G. (2009). Preferred reporting items for systematic reviews and meta-analyses: the PRISMA statement. *Annals of Internal Medicine*, *151*(4), 264–269. doi: 10.7326/0003-4819-151-4-200908180-00135 19622511

[CIT0040] Murad, M. H., Elamin, M. B., Garcia, M. Z., Mullan, R. J., Murad, A., Erwin, P. J., & Montori, V. M. (2010). Hormonal therapy and sex reassignment: A systematic review and meta‐analysis of quality of life and psychosocial outcomes. *Clinical Endocrinology*, *72*(2), 214–231. doi: 10.1111/j.1365-2265.2009.03625.x 19473181

[CIT0041] Nobili, A., Glazebrook, C., & Arcelus, J. (2018a). Quality of life of treatment-seeking transgender adults: A systematic review and meta-analysis. *Reviews in Endocrine and Metabolic Disorders*, *19*(3), 199–220. doi: 10.1007/s11154-018-9459-y 30121881 PMC6223813

[CIT0042] Nobili, A., Glazebrook, C., Bouman, W. P., Glidden, D., Baron-Cohen, S., Allison, C., Smith, P., & Arcelus, J. (2018b). Autistic traits in treatment-seeking transgender adults. *Journal of Autism and Developmental Disorders*, *48*(12), 3984–3994. doi: 10.1007/s10803-018-3557-2 29654452 PMC6223809

[CIT0043] Operario, D., Soma, T., & Underhill, K. (2008). Sex work and HIV status among transgender women: systematic review and meta-analysis. *Jaids Journal of Acquired Immune Deficiency Syndromes)*, *48*(1), 97–103. doi: 10.1097/QAI.0b013e31816e3971 18344875

[CIT0044] Owen-Smith, A. A., Gerth, J., Sineath, R. C., Barzilay, J., Becerra-Culqui, T. A., Getahun, D., Giammattei, S., Hunkeler, E., Lash, T. L., Millman, A., Nash, R., Quinn, V. P., Robinson, B., Roblin, D., Sanchez, T., Silverberg, M. J., Tangpricha, V., Valentine, C., Winter, S., … Goodman, M. (2018). Association between gender confirmation treatments and perceived gender congruence, body image satisfaction, and mental health in a cohort of transgender individuals. *The Journal of Sexual Medicine*, *15*(4), 591–600. doi: 10.1016/j.jsxm.2018.01.017 29463478 PMC5882508

[CIT0045] Pfeffer, C. A. (2008). Bodies in relation—Bodies in transition: Lesbian partners of trans men and body image. *Journal of Lesbian Studies*, *12*(4), 325–345. doi: 10.1080/10894160802278184 19042743

[CIT0046] Pistrang, N., Barker, C., & Humphreys, K. (2008). Mutual help groups for mental health problems: A review of effectiveness studies. *American Journal of Community Psychology*, *42*(1-2), 110–121. doi: 10.1007/s10464-008-9181-0 18679792

[CIT0047] Platt, L. F. (2020). An exploratory study of predictors of relationship commitment for cisgender female partners of transgender individuals. *Family Process*, *59*(1), 173–190. doi: 10.1111/famp.12400 30311216

[CIT0048] Platt, L. F., & Bolland, K. S. (2017). Trans* partner relationships: A qualitative exploration. *Journal of GLBT Family Studies*, *13*(2), 163–185. doi: 10.1080/1550428X.2016.1195713

[CIT0049] Platt, L. F., & Bolland, K. S. (2018). Relationship partners of transgender individuals: A qualitative exploration. *Journal of Social and Personal Relationships*, *35*(9), 1251–1272. doi: 10.1177/0265407517709360

[CIT0050] Riggs, D. W., von Doussa, H., & Power, J. (2015). The family and romantic relationships of trans and gender diverse Australians: An exploratory survey. *Sexual and Relationship Therapy*, *30*(2), 243–255. doi: 10.1080/14681994.2014.992409

[CIT0051] Roberts, A. L., Rosario, M., Slopen, N., Calzo, J. P., & Austin, S. B. (2013). Childhood gender nonconformity, bullying victimization, and depressive symptoms across adolescence and early adulthood: an 11-year longitudinal study. *Journal of the American Academy of Child & Adolescent Psychiatry*, *52*(2), 143–152. doi: 10.1016/j.jaac.2012.11.006 23357441 PMC3635805

[CIT0052] Simon, R. W. (2002). Revisiting the relationships among gender, marital status, and mental health. *American Journal of Sociology*, *107*(4), 1065–1096. doi: 10.1086/339225 12227382

[CIT0053] Soons, J. P., Liefbroer, A. C., & Kalmijn, M. (2009). The long‐term consequences of relationship formation for subjective well‐being. *Journal of Marriage and Family*, *71*(5), 1254–1270. doi: 10.1111/j.1741-3737.2009.00667.x

[CIT0054] Stewart, L., O'Halloran, P., & Oates, J. (2018). Investigating the social integration and wellbeing of transgender individuals: A meta-synthesis. *International Journal of Transgenderism*, *19*(1), 46–58. doi: 10.1080/15532739.2017.1364199

[CIT0055] Theron, L., & Collier, K. L. (2013). Experiences of female partners of masculine-identifying trans persons. *Culture, Health andSexuality*, *15*(sup1), 62–75. doi: 10.1080/13691058.2013.788214 PMC3696033 23668602

[CIT0056] Tong, A., Sainsbury, P., & Craig, J. (2007). Consolidated criteria for reporting qualitative research (COREQ): A 32-item checklist for interviews and focus groups. *International Journal for Quality in Health Care*, *19*(6), 349–357. doi: 10.1093/intqhc/mzm042 17872937

[CIT0057] White-Hughto, J. M., Reisner, S. L., & Pachankis, J. E. (2015). Transgender stigma and health: A critical review of stigma determinants, mechanisms, and interventions. *Social Science & Medicine*, *147*, 222–231. doi: 10.1016/j.socscimed.2015.11.010 26599625 PMC4689648

[CIT0058] World Health Organization. (2017). *International classification of functioning, disability and health.* http://apps.who.int/classifications/icfbrowser/

